# The Reentry and Disability Application Experiences of Formerly Incarcerated Older Adults

**DOI:** 10.1093/geroni/igaf122.1520

**Published:** 2025-12-31

**Authors:** Christian González-Rivera, Ruth Finkelstein

**Affiliations:** Brookdale Center for Healthy Aging, Hunter College, CUNY, New York, New York, United States; Hunter College, New York, New York, United States

## Abstract

This study investigates barriers that formerly incarcerated older adults (FIOA) face when applying for Social Security Disability Insurance (SSDI) and Supplemental Security Income (SSI), which are among the most important benefits that they rely on when integrating into society after prison. Justice-involved older adults often struggle to access these benefits due to systemic inefficiencies, inadequate pre-release assistance, and misinformation. Using a community-based participatory research approach, we conducted semi-structured interviews with 28 FIOA aged 55-75 and two key informants from reentry service organizations in New York State. Interviews explored participants’ experiences with Social Security Administration (SSA) processes, employment barriers, and interactions with agency personnel. Our findings revealed a range of structural, personal, and interpersonal hurdles: respondents frequently lacked essential documentation upon release, received inconsistent guidance from SSA staff, and experienced prolonged application delays averaging six months to a year. Many felt that SSA employees distrusted their disability claims, perceived discrimination based on their race or incarceration history, and faced digital barriers due to lack of knowledge about technology. Participants who received advocacy support from reentry organizations experienced smoother application processes. The study’s findings suggest a need for implementing pre-release benefit application assistance, enhanced SSA staff training, expanded support from advocacy organizations, trauma-informed reentry services, and clearer guidance on eligibility and documentation requirements. These implications may lead to policy and practice changes that would smooth the transition of older adults—especially those who served long sentences—from incarceration to community life.

